# Integrated omic profiling of the medicinal mushroom *Inonotus obliquus* under submerged conditions

**DOI:** 10.1186/s12864-023-09656-z

**Published:** 2023-09-19

**Authors:** Jinghua Hao, Xiaoli Wang, Yanhua Shi, Lingjun Li, Jinxin Chu, Junjie Li, Weiping Lin, Tao Yu, Dianhai Hou

**Affiliations:** 1 School of Bioscience and Technology, Weifang Medical University, Weifang, 261053 China; 2 School of Modern Agriculture and Environment, Weifang Institute of Technology, Weifang, 261053 China

**Keywords:** *Inonotus obliquus*, PacBio Sequel II sequencing, Widely targeted metabolomics, Secondary metabolites, Polyphenols

## Abstract

**Background:**

The *Inonotus obliquus* mushroom, a wondrous fungus boasting edible and medicinal qualities, has been widely used as a folk medicine and shown to have many potential pharmacological secondary metabolites. The purpose of this study was to supply a global landscape of genome-based integrated omic analysis of the fungus under lab-growth conditions.

**Results:**

This study presented a genome with high accuracy and completeness using the Pacbio Sequel II third-generation sequencing method. The *de novo* assembled fungal genome was 36.13 Mb, and contained 8352 predicted protein-coding genes, of which 365 carbohydrate-active enzyme (CAZyme)-coding genes and 19 biosynthetic gene clusters (BCGs) for secondary metabolites were identified. Comparative transcriptomic and proteomic analysis revealed a global view of differential metabolic change between seed and fermentation culture, and demonstrated positive correlations between transcription and expression levels of 157 differentially expressed genes involved in the metabolism of amino acids, fatty acids, secondary metabolites, antioxidant and immune responses. Facilitated by the widely targeted metabolomic approach, a total of 307 secondary substances were identified and quantified, with a significant increase in the production of antioxidant polyphenols.

**Conclusion:**

This study provided the comprehensive analysis of the fungus *Inonotus obliquus*, and supplied fundamental information for further screening of promising target metabolites and exploring the link between the genome and metabolites.

**Supplementary Information:**

The online version contains supplementary material available at 10.1186/s12864-023-09656-z.

## Background

The traditional medicinal mushroom *Inonotus obliquus* (*I. obliquus*), also commonly known as chaga, is a plant parasitic white-rot/brown-rot fungus, which belongs to the family *Hymenochaetaceae* of the phylum Basidiomycota [1, 2]. This medicinal fungus is widely distributed in North America, Asia, and Northern Europe, and has been used for more than four centuries as a folk medicine in Northern Europe for the treatment of stomach diseases, intestinal worms, liver and heart ailments [2–7]. Pharmacological studies on the bioactive substances of the fungus in cancer research have been demonstrated its excellent medicinal value in the treatment of various human tumors [5, 8–13], and its powerful effect in the treatment of several human diseases without any unacceptable toxic side effects, has become demonstrated [14, 15].

Previous chemical investigations of extracts from the fruit body and submerged cultures of *I. obliquus* have demonstrated that the fungus produces multiple types of bioactive components, including polysaccharides, organic acids, phenoliccompounds, terpenoids, ligins and melanins [2, 4, 5, 8, 9, 16–19]. More than 100 species of metabolites have been identified, most of which are involved in antioxidation, antitumor and immunomodulation activities [5, 20]. Nevertheless, most studies have focused on metabolites in the fruit body of *I. obliquus* [5, 21, 22]. Due to the slow growth of *I. obliquus* in its natural habitat [2, 5], submerged cultures of this fungus have been focused on the identification of bioactive secondary metabolites [20, 23–26], and 26 phenolic compounds have been identified in the mycelia and categorized into small phenolics, glycosylated flavonoids (GF), flavonoid aglycones (FAG) and polyphenols [20]. Furthermore, genomic sequencing of the fungus [27] and differential transcriptomic analysis of submerged cultures of the fungus under different culture condition has been applied to identify and understand the regulation of genes involved in secondary metabolite biosynthesis, especially terpenoid biosynthesis [28].

In previous studies, high-performance liquid chromatography (HPLC) and nuclear magnetic resonance (NMR) spectroscopy have been used to rapidly advance studies of chemical composition and quality evaluation of metabolites of the fungus [5, 12]. Recently, HPLC-MS (mass spectrometry) based metabolomics has been increasingly applied in discovery studies for comprehensive quality assessment of multiple metabolites [29, 30]. In addition, widely targeted metabolomics is a promising novel technique for large-scale, ultrasensitive qualitative and quantitative analysis of targeted metabolites of interest [31], and facilitates the understanding of metabolic pathways contributed to the modulation of metabolites in fungi [29, 32–35], plants [36, 37] and animals [38].

In this study, we presented the genome of the *I. obliquus* (strain CFCC 83,414), and further integrated comparative omic analysis of the fungus under different submerged culture conditions. Our results identified multiple secondary metabolites and elucidated an understanding of the regulations of metabolite productions at transcriptomic, proteomic metabolomic levels.

## Results

### Genome assembly and inference of chromosomes

We sequenced genomic DNA of the *I. obliquus* using PacBio Sequel II and Illumina platforms, yielding high-quality data of ~ 64× coverage (2.32G, PacBio platform) and ~ 244× coverage (8.82G, Illumina platform), respectively (Table S1 and S2). The size of the assembled genome of the *I. obliquus* was 36.13 Mb, including 32 contigs with an N90 of 1.94 Mb and a GC content of 47.39% (Table 1). The heterozygous ratio was estimated to be 0.82%. The completeness of the predicted protein-coding gene set was estimated to be 95.2% complete based on the fungal BUSCO families (Table S3).

**Table 1:** Characteristics of the assembled contigs and genome of the *Inonotus obliquus* CFCC 83,414

Assembly	Iterm	Statistics
Contig	Total number Total length (bp) N50 (bp) N90(bp) Max length (bp) GC content (%)	32 36,127,648 3,182,639 1,935,300 4,357,162 47.39
Genome	Genome assembly (bp) Number of protein coding genes Average length of protein-coding genes (bp) Average length of protein-coding sequences (bp) GC content of protein-coding sequences (%) Average number of exons per gene Total length of repeat size (bp) No. of tRNAs No. of rRNAs	36,127,648 8352 2271.13 1710.55 50.77 7.08 7,338,030 180 28

**Fig. 1:**
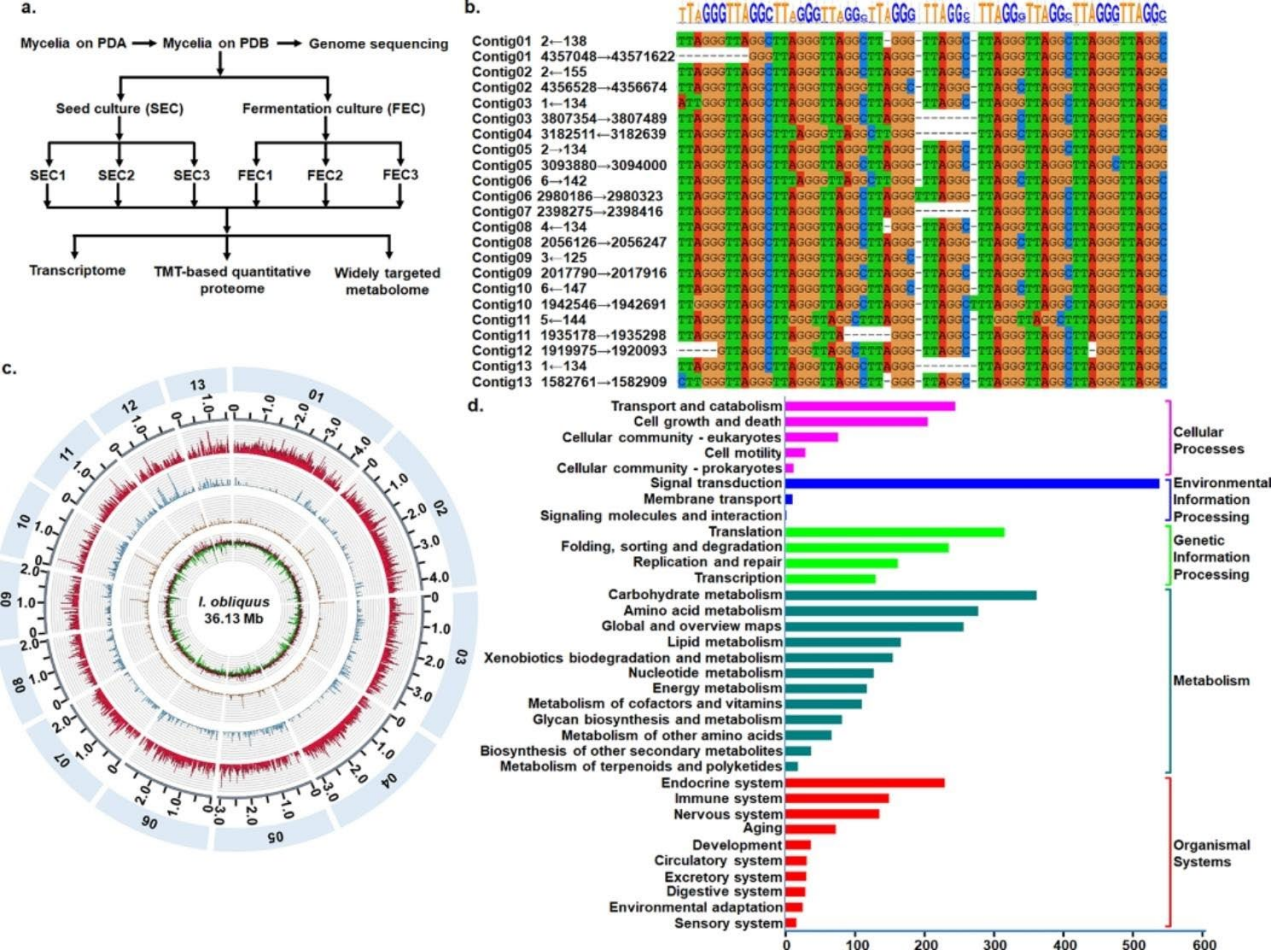
Assembly and annotation of the genome of *I. obliquus.* (**a**) Experimental schema of multiple omic analysis. (**b**) Alignment of the predicted telomeric tandem repeat sequences at terminals of 13 major contigs. The start-end sites of telomere tandem repeat sequences were indicated following the contig names; (**c**) Characterization of 13 major contigs of the genome. From the inner to the outer ring in order is the GC content in each contig, repeat sequence density in each contig, transposable element density in each contig, and gene density in each contig; (**d**) The KEGG functional annotation of genes. The gene number was noted at the right of the bar corresponding to relevant class, and the bar length indicated the gene number

Among the 32 contigs, 13 major contigs (approximately 1.58–4.36 Mb in length) compresed 98.63% of the entire genome, while the remaining 19 minor contigs (approximately 15.40–55.10 kb in length) (Table S4) comprised 1.37% of the genome. The repeat sequences mostly proximal to both terminals of these 13 major contigs were collected and it was found that approximately 18–24 repeat units containing 6 bases of TTAGGG(C) at the 5’- end of 10 contigs with the exception of the contig 4, 6 and 7) and the 3’- end of all the 13 contigs (Fig. 1b). The sequence of TTAGGG(C) was highly similar to the telomere tandem repeat sequence frequently found in various species of invertebrates [39–41], plants [42], fungi [41, 43] and so on [41, 44] (see details in http://telomerase.asu.edu/sequences_telomere.html), and was considered to be the telomeric tandem repeat sequence of the *I. obliquus* in this study. It seemed that the karyotypes of *I. obliquus* appeared to be 12–13 chromosome pairs. Detailed characteristics of the 13 large contigs were shown in Fig. 1c.

### Genome annotation of the Inonotus obliquus

The genome annotation was performed using ab initio prediction, homology-based searches as well as a cDNA-based evidential support of transcriptional data in this study. A total of 8352 protein-coding genes were predicted with a length of 2271 bp and 7.08 exons per gene on average, of which, 8347 genes (≥ 99.9%) were located on the 13 major contigs. Further, 7915 genes (94.8%) were annotated (Table S5) and 3885 (46.5%) genes were functionally annotated according to the KEGG annotation (Fig. 1d). In addition, a total number of repetitive sequences and non-coding RNAs accounted for 20.31% and 0.4% of the genome respectively.

Further, a total of 365 CAZyme-coding genes, including 72 auxiliary activities (AAs), 3 carbohydratebinding modules (CBMs), 21 carbohydrate esterases (CEs), 187 glycoside hydrolases (GHs), 69 glycosyltransferases (GTs) and 13 polysaccharide lyases (PLs) (Table S6) were identified in the genome. The CAZyme-coding genes were categorized into several superfamilies, among which, GHs, GTs and AAs were larger superfamilies than the others, and the numbers of GHs were significantly larger than the numbers of other categories, consistent with the saprophytism lifestyle of using lignocellulose decomposition. The biosynthetic genes of secondary metabolites were usually found to be clustered [45], and due to secondary metabolites have noteworthy pharmaceutical potential and the absence of risks to human and animal health, genomic analyses have provided useful information for elucidating the biosynthesis of secondary metabolites, such as terpenoids, polyketides, and nonribosomal peptides [46–50]. In the genome, a total of 19 BGCs were predicted (Table S7), including 2 hybrid (mixed NRPS-like and type 1 PKS) BGCs, 1 NRPS, 1 type 1 PKSs, 3 NRPS-like BGCs, and 12 BGCs encode for terpenes including the antitumor compound clavaric acid [51, 52].

### Overview of the Inonotus obliquus transcriptome

**Fig. 2:**
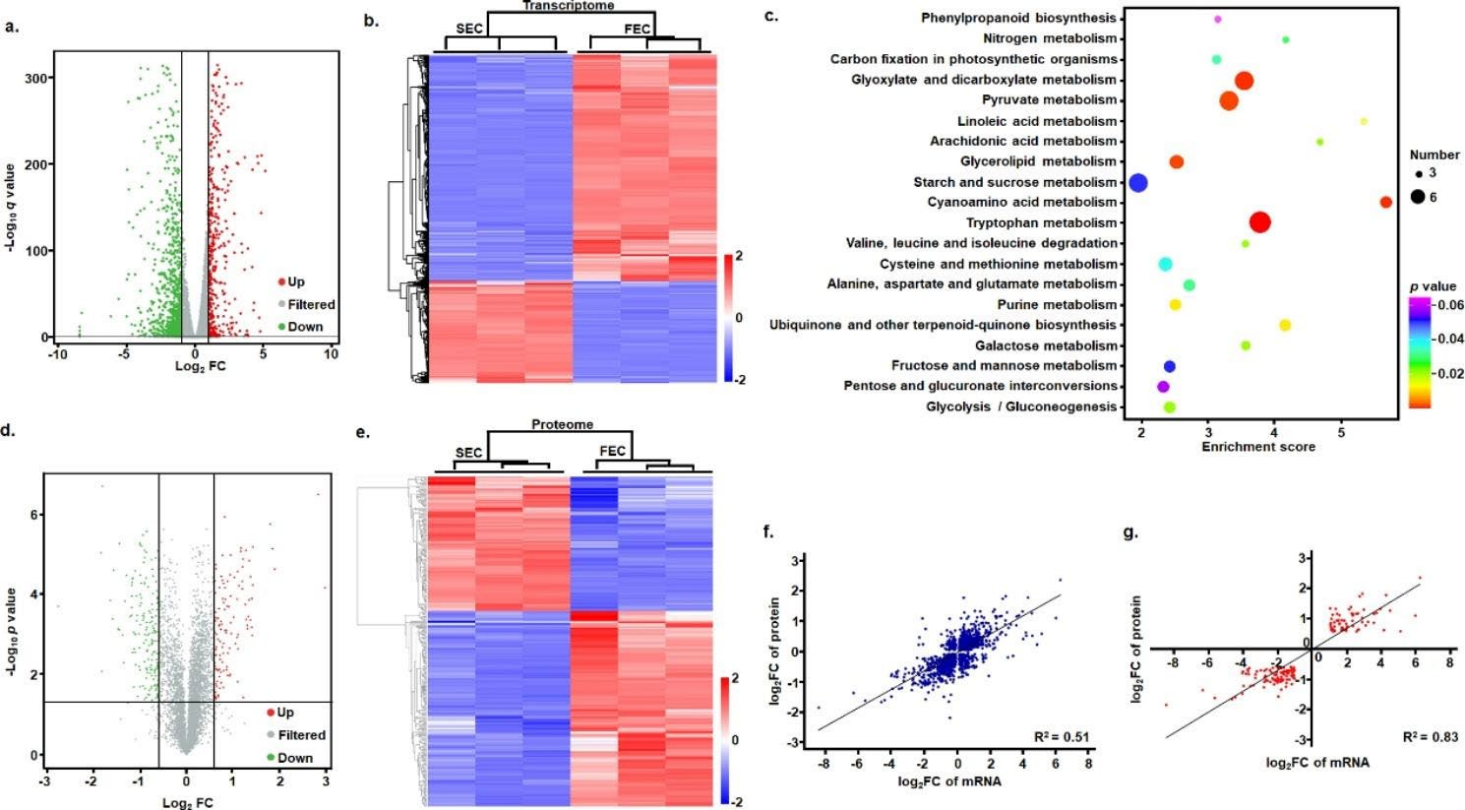
Transcriptome and proteom analysis of the *I. obliquus* under the seed culture (SEC) and fermentation culture (FEC) conditions. (**a**) Volcano plot showing differential transcription of genes; (**b**) Transcrtiption heatmap; (**c**) Pathway enrichment in FEC vs. SEC; (**d**) Volcano plot showing differential expression of proteins; (**e**) Exprssion heatmap; (**f**) Correlation between the mRNA and protein levels of 1648 differential genes; (**g**) Correlation between the mRNA and protein levels of 157 differential proteins of group I

For the triplicate seed culture (SEC) and fermentation culture (FEC) samples, a total of 42.18 G of clean data were acquired from transcriptomic sequencing, with an arrange of 6.5 ~ 7.38 G for each sample and an average GC content of 50.79% (Table S8). Approximately 96.93 ~ 97.07% of the expressed sequence tag sequences (EST) generated from RNA-Seq data were mapped to the genome referred, indicating almost complete coverage of the protein-coding gene regions (Table S9). Further analysis showed that 8149 and 8117 of the whole 8352 genes in the genome were covered under the SEC and FEC environment, respectively (Table S10). Candidate differential genes involved in FEC adaptation were chosen from 8049 quantified genesusing uniquely mapped reads in both environment (Table S10). The transcripts of 1283 genes were detected to be associated with FEC adaptation, including 399 up-regulated genes and 884 down-regulated genes (Fig. 2a). The differentially expressed genes (DEGs) showed distinct transcriptional patterns under different culture conditions, which was depicted intuitively in Fig. 2b with the hierarchical clustering of DEG transcription levels. Furthermore, for all the fold changes of all up- and down-regulated genes, 69.8% of DEGs were altered 2–4-fold at the mRNA level, while fewer DEGs (18.5%) were altered more than 4–8-fold, and only 11.7% of DEGs were altered more than 8-fold. Among these DEGs, many were related to fungal metabolic functions, such as Aldo/keto reductase (76.39-fold), terpenoid synthase (22.96-fold), and cyclopropane fatty acid synthase (12.12-fold). The functional classification of DEGs was performed by mapping to the KEGG (Kyoto Encyclopedia of Genes and Genomes) pathway, demonstrating that the DEGs were mainly associated with the biosynthesis and metabolism of various metabolites including tryptophan, cyanoamino acid, sucrose and purine (Fig. 2c), and also with some transport and catabolism, signal transduction and membrane transport pathways. In general, the results implied that a considerable portion of genes involved in various cellular and physiological processes were strongly affected at the transcriptional level following FEC condition adaptation.

### TMT-based quantitative proteome and its correlation with transcriptome

Proteins were extracted from the triplicate SEC and FEC samples and subjected to TMT (tandem mass tag)-based proteomic analysis. A total of 4962 proteins were identified, among which, 4729 proteins were quantified with at least one peptide and 4265 were quantified with at least two peptides (Table S11). A total of 264 differential expression proteins (DEPs) was detected to be associated with FEC adaptation, including 105 up-regulated genes and 159 down-regulated proteins (Fig. 2d), which were depicted intuitively with the hierarchical clustering of DEP expression levels in Fig. 2e. For the fold changes in DEPs of all up- and down-regulated genes, 75.4% of DEPs were altered 1.5–2-fold at the protein level, whereas fewer DEPs (20.8%) were altered more than 2–3-fold, and only 3.8% of DEGs were altered more than 3-fold, which were still less than 6-fold, demonstrating lower fold changes at expression level than at transcription level of of differential genes.

To examine whether a stronger correlation exists between the mRNA and protein level changes after the adaptation to fermentation condition, combination of transcriptomic and proteomic analyses were performed using linear regression analysis based on the log2-transformed gene fold changes for pair-wise comparison of the TMT-based quantitative proteomic (Table S11) and RNA-Seq results (Table S10). For all proteins identified in TMT-based quantitative proteomic analysis, the correlation coefficient between the mRNA and protein level changes was only 0.31, whereas, that of all the data of differential 1608 genes (including all the 264 DEPs in quantitative proteome) with *p* value ≤ 0.05 at transcription level and with *p* value ≤ 0.05 at expression level was 0.51(Fig. 2f). There was a positive correlation (0.61) between mRNA and protein level changes for the 264 DEPs, showing similar trends at both of the mRNA and protein levels after of FEC-condition adaptation. Further, all the 264 DEPs of the TMT-based quantitative proteome were categorized into three groups based on the following patterns: group I (including 157 DEPs) (Table S12), the mRNA and protein levels showed the same changes; group II (including 104 DEPs), mRNAs were basically unchanged while protein levels were up- or down-regulated; and group III (including 3 DEPs), the directions of mRNA and protein changes were opposite. And for the 157 DEPs of group I, the correlation coefficient between the changes at mRNA and protein levels was 0.83 (Fig. 2g), indicating a strong positive correlation with their mRNA in expression patterns. These proteins were involved predominantly in metabolism-related physiological process, including proteins involved in polysaccharide, carbohydrate, amino acid, lipid and purine metabolism, transmembrane transpor, and also in protein phosphorylation, and signaling molecules and interaction (Table S12).

### Identification anf quantatition of secondary metabolites

**Fig. 3:**
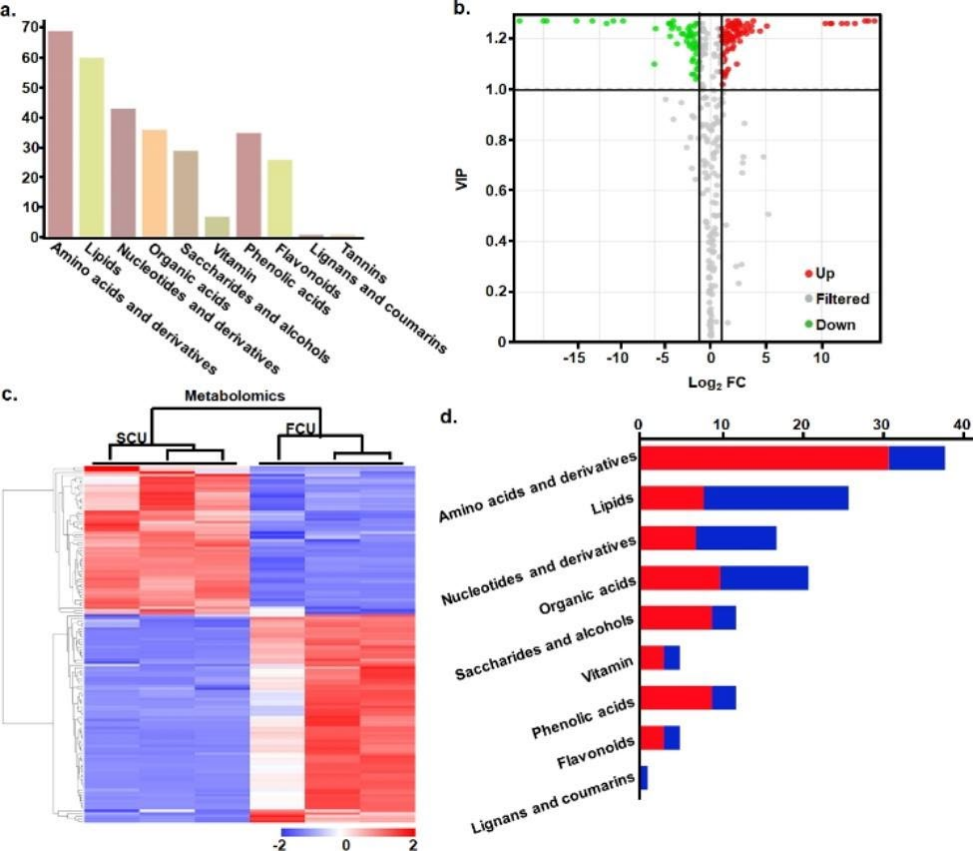
Widely target metabolomic analysis of the *I. obliquus* under the seed culture (SEC) and fermentation culture (FEC) conditions. (**a**) Classification of identified 307 secondary metabolites; (**b**) Volcano plot showing differential production of metabolites; (**c**) Production heatmap of 157 differential production of metabolites; (**d**) Classification of 157 differential production of metabolites. Red: Up-regulated; Blue: down-regulated

We further focused on production of secondary metabolites using widely targeted metabolomics. The metabolomic profiling of *I. obliquus* under SEC and FEC conditions was performed using HPLC-MS/MS identification, and led to a comprehensive identification and quantification of a range of 307 metabolites, including 68 amino acids and derivatives, 60 lipids, 44 nucleotides and derivatives, 36 organic acids, 35 phenolic acids, 29 saccharides and alcohols, 7 vitamins and 26 flavonoids, and a few of lignans and coumarins and tannins (Fig. 3a and Table S13). Significantly regulated metabolites between the two condition groups were determined with VIP value ≥ 1 and log2 FC value ≥ 1 or ≤ -1 (Fig. 3b) and hierarchical cluster analysis (HCA) was performed (Fig. 3c). A total of 137 differential metabolites were selected, among which, 59 metabolites were more productive and 43 metabolites were less productive under FEC condition than under SEC condition (Fig. 3d). For the fold changes in differntial generated metabolites (DGMs), 100 (73%) of DGMs were altered 2–8 fold at the metabolite level, whereas 37 (27%) were altered more than 8-fold, among which, 17 metabolites including amino acids and derivatives, phenolic acids, flavanols and organic acids were altered more than 100 fold, demonstrating a remarkable change at metabolite level.

## Discussion

This study described the assembly and annotation of the genome of the *I. obliquus* (strain CFCC 83,414), and provided a comprehensive view on the response of the fungus to the changes in submerged culture conditions at transcriptomic, proteomic and metabolomic levels through multi-omic analysis based on the sequenced genome. Our results demonstrated differences in metabolism-related pathways between seed culture and fermentation culture conditions and shed light on understanding of the regulation of secondary metabolite productions.

For basidiomycetes, the karyotype had been found to be conserved, with the number of known karyotypes mainly ranging from 11 to 14 chromosome pairs [53–55]. The genome of *I. obliquus* presented in this study was sequenced using the PacBio Sequel II HiFi sequencing technology with high coverage and accuracy [56]. Considering the analysis of telomeric tandem repeat sequence, it seemed that the genome contains 10 completed chromosome-length contigs in the 13 major contigs and the remaining 19 minor contigs (total length < 0.5 Mb), and the karyotype of *I. obliquus* would be 12–13 chromosome pairs. The higher BUSCO rate of 95.2% compared to that (about 90%) of the previous reported genome of the *I. obliquus* strain CT5 which the sequenced using the Oxford Nanopore PromethION sequencing platform [27], also suggested the high-quality mononuclear genome assembled in this study. Further construction of chromosome-level genome map using the Hi-C protocol [57, 58] would be used to understand the detail genome organization and future comparative genomic analysis of more genomes of *I. obliquus* stains and other speices.

After the transformation of the mycelia from the seed culture into fermentation culture, various biological processes were differentially regulated, resulting in the differential change of the production of secondary metabolites. The RNA-seq based comparative transcriptome yielded much more comprehensive and detailed information about transcriptomic profiling (8049 quantified genes) than the comparative proteome (with 4729 proteins quantified) which demonstrated lower fold changes of differential genes at expression level than at transcription level. To explore the consistency between mRNA and protein levels, correlation analysis was performed for all the genes quantified at both transcriptional and translational levels, and the results suggested a positive association but rather poor (0.51). Nevertheless, a strong positive correlation (0.83) was found between changes at the mRNA and protein levels for the 157 DEPs of group I among all the 264 DEPs, which gived insight into investigating how reliably the transcriptional profile reflects the translational profile. Further studies would be performed to explore the multiple-time-point dynamic changes [20] of production pathways of secondary metabolites under fermention condition rather a single time point experimental design.

The widely targeted metabolomics method based on the LC-MS/MS technology was a very sensitive and accurate method for the measurement of targeted metabolites, which was facilitated by the construction of MS2 spectral tag (MS2T) libraries [31]. Taken into considerartion the high sensitive detection capability of widely targeted metabolomics and the ambiguity that metabolites from the sclerotium may be self-sunthesized or acquried from the hosts or culture mediums with undefied compositions, the fungus grown on the FEC cunlture condition with chemically defined medium was selected with that grown on the SEC culture condition for identifcation and quantification of secondary metabolites synthesized by the fungus *I. obliquus*. This identification and quantification of a series of 307 secondary metabolites supplied comprehensive information on fungal secondary metabolites under fermentation conditions, significantly updating the list of potential bioactive metabolites [5]. Due to the close relationship of biosynthetic pathways [59], many bioactive polyphenols including flavonoids and phenolic acids, such as caffeic acid, vanillic acid, isorhamnetin-3-O-arabinoside, 3’,4’,7-trihydroxyflavone were found to be highly accumulated in fermentation culture, which is in consistent with previous studies [20, 23, 24]. The accumulation of multiple bioactive polyphenols including flavonoids and phenolic acids may suggest that some proteins including the phenylalanine ammonia lyase (PAL) and cinnamate 4-hydroxylase (C4H, a cytochrome P450 monooxygenase) that are located upstream of the two biosynthetic pathways for basic structural skeletons of flavonoid and phenolic acid compounds, will be upregulated. It has been demonstrated that an increased expression of PAL can lead to the accumulation of flavonoids [60]. Furthermore, among the 157 differentially expressed genes/proteins in group I, we found that the gene001065, encoding a cytochrome P450 monooxygenase protein with a C4H domain (with *E* value of 1.54e-46) (ID in NCBI: PLN02394), was upregulated with a 5.12-fold change in transcription level and a 2.17-fold change in expression level, suggesting its correlation with the accumulation of flavonoid and phenolic acid compounds. The biosynthetic pathway of flavonoid compounds seems to be present in the fungus, although the chalcone synthase (CHS) responsible for producing the key precursor of flavonoid compounds is still not found in the genomes of the fungus and other mushrooms [61]. This may facilitate the elucidation of the regulation of production of bioactive polyphenols and promote further reseaches on identification of novel CHS-like genes with homology or functional similarity.

Neverthelss, terpene synthases were found to be up-regulated at both the transcriptional and translational levels, terpenes were not found here. In addition, aqueous alcohols can capture a broad spectrum of metabolites but mostly on the polar side. Some constituents of intermediate polarity and non polar metabolites may be under-presented in the LC-MS/MS data. Hence, the variation between the two types of culture, in terms of non-polar metabolites, is left out in this study. For further comprehensive identification of metabolites, especially that from the sclerotium, it is suggested to use a combination of different solvents for metabolite extration to improve the identification coverage, as well as additional corresponding MS-based detection, such as GC (gas chromatography)-MS/MS detection. It is noticed that due to the absence of environmental stimuli, fewer secondary metabolites accumulated under fermentation conditions compared to those of fungi grown in natural habitats, highlighting that the large proportion of potentially bioactive compounds are waiting to be identified. Further efforts will be needed to use a variety of stimuli to promote the productions of secondary metabolites and use the *I. obliquus* as a reliable source for pharmaceutical purposes [24, 25, 62–67]. Furthermore, in combination with this study, further transcriptomic analysis and comprehensive identification of additional metabolites of the fungal sclerotium will promote further exploring the link between sequenced fungal genomes and bioactive fungal secondary metabolites [47]. In combination with bioinformatic tools, construction of genetic engineered fungi through knock-out of target genes [47, 68, 69] will be conducted for identification of genes involved synthesis pathways and discovery of novel fungal secondary metabolites [70].

In summary, genome sequencing, integrated comparative omic approach and metabolomic profiling revealed the genetic basis of metabolic profile of the fungus *I. obliquus* under SEC and FEC conditions. The large number of secondary metabolites self-synthesized by the fungus with widely targeted profiling and quantification supplied fundamental information for further screening of promising target metabolites under fermentation conditions. In the future, this multi-omic should assist the scientific community in genetic manipulation and metabolic engineering for the production of potential phamaceutical metabolites based on the presented genome with high accuracy.

## Methods

### Preparation of Fungal materials for multiple omics

The culture of *I. obliquus* (strain CFCC 83,414) was purchased from the China Forestry Culture Collection Center (CFCC) and maintained on potato dextrose agar (PDA) culture medium (Solarbio, China) at 26℃ in darkness. The mycelia were transferred to PDB culture medium (Solarbio, China) and incubated at 26℃ and 150 rpm for 72 h as previously described [20]. The mycelia were then homogenized and inoculated aseptically [20] into 500 ml conical flasks containing 150 ml PDB medium (used as seed culture in this study and named SEC) and the medium (used as fermentation culture in this study and named FEC) consisting of 2% glucose, 0.35% peptone, 0.01% KH_2_PO_4_ and 0.05% MgSO_4_·7H2O. The culture was incubated at 26℃ and 150 rpm for 7 days, respectively, and used for further differential analysis of transcriptomics, proteomics and widely targeted metabolomics (Fig. 1a) and three biological triplactes were performed (Fig. 1a).

### Genome sequencing, assembly and annotation

The genomic DNA of the mycelia was prepared as previously described [71–73]. The genomic DNA was sequenced with the Illumina HiSeq X Ten and the PacBio Sequel II sequencing platform using the Circular Consensus Sequencing (CCS) model [74] from Biomarker Technologies Co., Ltd (Beijing, China) and the obtained data were further processed according to the conventional Biomarker pipeline. Briefly, the initial PacBio Sequel II sequence data was processed with the SMRT LINK (https://www.pacb.com/support/software-downloads/) (v10.0) to obtain consensus reads (high fidelity reads, HiFi reads). The HiFi reads obtained with an N50 of > 11 kb and accuracy of > 99% were assembled using Hifiasm [75] (v0.15.5) and further curated using Pilon [76] (v1.23) for the primary assembly using the Illumina-derived short reads generated above [72] to correct any remaining errors.

The prediction of gene structure was performed through a combination of homology-based prediction, transcriptome-based prediction and *de novo* prediction methods as follows. Firstly, the protein sequences of four *Hymenochaetaceae* genomes (*Fomitiporia mediterranea*, *Phellinidium pouzarii*, *Pyrrhoderma noxium* and *Sanghuangporus baumii*) were aligned to the assembly using TblastN [77] and the gene structure of the corresponding genomic regions for each BLAST hit was predicted using GeneWise [78] (v2.4.1). Secondly, the gene structure was predicted with Transdecoder [79] (v3.01) based on transcripts assembled from differential transcriptome analysis using Trinity [79, 80] (v2.3.2), while cufflinks [81] (v2.2.0) was then used to assemble the transcripts into gene models which also used for the further ab initio prediction. Thirdly, the ab initio gene prediction was from the repeat-masked genome using Augustus [82] (v3.3.3) and GeneMark [83] (v4.33) with default parameters. All predicted genes from the above forecast results were combined into a non-redundant set of gene structures using EVidenceModeler [84] (EVM, v1.1.1).

For the prediction of noncoding RNAs (ncRNAs), the tRNAs and rRNA were predicted using tRNAscan-SE(v1.3.1) [85] and RNAmmer [86] (v1.2), respectively, while small nuclear RNA (snRNA) and small nucleolar RNA (snoRNA) sequences were identified using Infernal [87] (v1.1.2) against the Rfam [88] (v14.0) database. For the analysis of repetitive sequences and transposable elements (TEs), homology-based prediction was performed using the RepeatMasker [89] (v4.0.7) against the repeated sequence database RepBase [90]. The ab initio prediction was performed using the RepeatModeler (http://www.repeatmasker.org/RepeatModeler/) for the establishment of de novo repeat sequence library and then the RepeatMasker [89] against the *de novo* repeat sequence database generated from the RepeatModeler’s prediction above. Furthermore, the tandem repeat sequences were searched in the genomic sequence using Tandem Repeat Finder (TRF) [91] (v4.09). In addition, for identification of telomere sequences, repeat sequences were aligned using MAFFT [92, 93] (v7.310) with the G-INS-i option, and the sequence logo of alignment sequences was generated using WebLogo [94].

Gene functions were inferred according to the best match of alignments using BLAST (e < 1e-5) against functional databases including GO [95], KEGG [96], COG/KOG [97], NR (https://ftp.ncbi.nlm.nih.gov/blast/db/), Swissprot and TrEMBL [98] and search of the database Pfam [99] (v35) using HMMER [100]. Annotation completeness analysis was performed using BUSCO (v3.1.0) [101] search against the dataset of fungi_odb9 (https://busco-archive.ezlab.org/v3/frame_fungi.html).

### Identification of CAZymes and secondary metabolite BCGs

The CAZymes in the genome of *I. obliquus* were identified using dbCAN2 meta server [102] with HMMER [100], DIAMOND [103] and eCAMI [104] search tools with default parameters and following the server‘s guidelines and recommendations (https://bcb.unl.edu/dbCAN2/help.php). Proteins were identified, grouped into CAZymes families defined in the CAZy database [105] and selected according to the recommendation of the dbCAN2 server. The biosynthetic gene clusters (BCGs) of secondary metabolites were predicted using AntiSMASH 6.0.1 [106] with all extra features selected.

***Transcriptomic analysis and identification of DEGs.***

Total RNA was extracted from each sample (triplicate SEC and FEC samples) using mirVana miRNA Isolation Kit (Thermo, USA) and 4 µg of total RNAs from each sample were used for construction of cDNA library using TruSeq Stranded mRNA LTSample Prep Kit (Illumina, USA) and then sequenced using Illumina HiSeq X Ten system. All the experiments were conducted following the manufacturers’ protocols.

The acquired raw reads in fastq format were firstly processed using Trimmomatic [107] (v0.36) and low quality reads were removed to obtain clean reads, then the retained clean reads were mapped to reference genome of *I. obliquus* using HISAT2 [108] (v2.2.1.0). The read counts of each gene were obtained using HTSeq [109] (v 0.6.0) and the gene FPKM (fragments per kilobase of transcript per million fragments mapped) [110] expression values were calculated using cufflinks [81] (v2.2.0). To calculate fold changes, the number of reads for each gene in each library was normalized by the total number of mapped reads for the library using the DESeq2 [111] (v1.20.0) R package functions estmateSizeFactors and nbinomTest. The *p* value (≤ 0.05, negative binomial test) as well as FC (fold change) value were further calculated using in DESeq2. Genes with a significant *p* value (≤ 0.05) and FC value (≥ 2 or ≤ 0.5) were considered as differentially expressed genes (DEGs). And the KEGG [96] pathway enrichment analysis of DEGs were performed based on the hypergeometric distribution.

### TMT-based quantitative proteomic analysis

The mycelia samples (SEC and FEC samples in triplicate) were frozen in liquid nitrogen and grinded into fine powder. Approximately 30 mg powder of each sample was mixed with 5 volumes (w/v) of TCA (trichloroacetic acid)/acetone (1:9) (Sigma, USA), vortexed, and then incubated at -20℃ for 4 h. The precipitates were centrifuged at 6000 g for 40 min at 4℃, and washed three times with pre-cooled acetone. After air-dried at room temperature, the pellets were solubilized with 30 volumes (w/v) of SDT lysis buffer (4% SDS, 100 mM Tris-HCl, pH 7.6) [112]. The respended lysates were incubated at 95℃ for 5 min, sonicated on ice, and then incubated at 95 °C for 15 min. The supernatants were collected through centrifugation at 14,000 g for 15 min, and filtrated using 0.22 μm filters (Millipore, USA). The protein concentrations of the resultant filtrates were determined using the bicinchoninic acid (BCA) assay kit (Beyotime, China). Approximately 100 µg proteins from each of all samples were reduced with a final concentration of 100 mM dithiothreitol (DTT) at 95℃ for 5 min, and subjected to trypsin digestion following the FASP protocol as previously described [112, 113] using 30 kDa Ultra filter unit (Sartorius, Germany). The desalted peptides were resuspended in 40 µL 0.1% formic acid (Thermo Fisher Scientific, USA) and measured at OD280 using Nano Drop 3000 spectrometer (Thermo Fisher scientific, USA).

The TMT (tandem mass tag)-labeling was performed by Shanghai Genechem Co., Ltd (China) following the product instruction of TMTsixplex Isobaric Label Reagent Set (Thermo Fisher scientific, USA) and as previously described [114]. All six samples from SEC and FEC samples in triplicate, each containing 100 𝜇g protein digest, were mixed with the TMT label dissolved in 41 µL anhydrous acetonitrile and incubated for 2 h at room temperature. The TMT labels 126, 127 and 128 were used for triplicate SEC samples, while the labels 129, 130 and 131 were used for triplicate FEC samples. The reaction was quenched by adding 8 µL of 5% hydroxylamine, and then all the labeled samples were combined and further separated on a 1260 infinity II HPLC System (Agilent, USA) equipped with an XBridge Peptide BEH C18 Column (130Å, 5 μm, 4.6 mm × 100 mm, Waters). The mobile phase consisted of two components, with component A being 5% acetonitrile (ACN) with 0.1% ammonium formate and component B being 85% ACN with 0.1% ammonium formate. The 85 min solvent gradient at a flow rate of 1 mL/min was set as follows: 0% B within 25 min, 0 − 7% B in 5 min, 7 − 40% B in 25 min, 40 − 100% B in 5 min and 100% B for 15 min. Fractions were collected every minute for a total of 40 fractions. All fractions were dried by vacuum centrifugation (Huamei, China), then reconstituted with 10% formic acid, and further combined into ten samples prior to LC − MS/MS analysis.

The MS data were acquired with an Easy nLC system (Thermo, USA) coupled to an Orbitrap Q-ExactiveTM Plus mass spectrometer (Thermo, USA). Peptides were trapped (Acclaim PepMap RSLC 50 𝜇m × 15 cm, nano viper, Thermo, USA) before being seperated on the Easy nLC system. The mobile phase consisted of two components, with component A being 0.1% formic acid in water and component B being 0.1% formic acid in 80% ACN. The 90 min solvent gradient at a flow rate of 300 nL/min was set as follows: 6% B within 5 min, 6 − 38% B in 70 min, 38 − 100% B in 10 min, and 100% B for 5 min. Full scan MS spectra from m/z 350–1800 were acquired at a resolution of 70,000 with automatic gain control (AGC) set to 3e6 and a maximum injection time (IT) set to 50 ms, followed by 10 MS2 scans of precursors selected for fragmentation by higher-energy collision dissociation (HCD) with normalized collision energy set to 30 ev. All MS2 spectra were acquired at a solution of 35,000 with a maximum injection time (IT) set to 45 ms.

Raw files in raw were transformed into files in mgf format using Proteome Discoverer (v2.2, Thermo, USA) and uploaded onto MASCOT (v2.6) server against the newly annotated protein database of of the *I. obliquus*. The search parameters as previously descried were used [112] and identified peptides were filtered to a 1% false discovery rate (FDR). Proteins that showed more than two-fold change (FC of ≥ 1.5 or ≤ 0.67) with *p* value ≤ 0.05 [115] were considered to show significant differential expression. Linear regression analysis was performed based on the log2-transformed fold changes of the TMT-based quantitative proteomic and RNA-Seq results for pair-wise comparison. Correlation was regarded as strong when R^2^>0.81 [116, 117].

### Extraction and quantification of metabolites

The mycelia from each of all samples (triplicate SEC and FEC) were freeze-dried, and then extracted by 70% aqueous methanol following the previously described [31]. The freeze-dried mycelia were crushed using a mixer mill (MM 400, Retsch, Germany) with a zirconia bead for 1.5 min at 30 Hz. The 100 mg powder was incubated with 0.6 ml 70% aqueous methanol overnight at 4 °C. The extracts were collected after centrifugation at 10, 000 g for 10 min, and then filtrated using 0.22 μm filter (SCAA-104, ANPEL, China). The MS data was acquired with an UPLC (Shim-pack UFLC SHIMADZU CBM30A system, SHIMADZU, Japan) coupled to a 4500 Q-TRAP tandem MS system (Applied Biosystems, USA). The extracts were separated by using a Shim-pack UFLC SHIMADZU CBM30A system, SHIMADZU, Japan). Metabolites of 4 µL of each extract were separated on the UPLC equipped with an SB-C18 (1.8 μm, 2.1 mm × 100 mm, Agilent, USA). The mobile phase consisted of two components, with component A being 0.1% ammonium formate and component B being 100% ACN. The 90 min solvent gradient at a flow rate of 0.35 mL/min was set as a program that employed the starting conditions of 95% A, 5% B. Within 9 min, a linear gradient to 5% A, 95% B was programmed, and a composition of 5% A, 95% B was kept for 1 min. Subsequently, a composition of 95% A, 5.0% B was adjusted within 1.10 min and kept for 2.9 min. The column oven was set to 40 °C. The effluent was alternatively connected to an ESI (electrospray ionization)-triple quadrupole-linear ion trap spectrometer (Q-TRAP). The ESI source operation parameters were set as previously described [31], whereas ion source gas I (GSI), gas II (GSII), curtain gas (CUR) were set at 50, 60, and 30.0 psi, respectively, and QQQ (triple quadrupole) scans were acquired as MRM (multiple reaction monitoring) experiments with collision gas (nitrogen) set to 5 psi [31]. Quality control (QC) samples were used and injected to the mass spectrum to monitor the repeatability of the analysis process.

The MS/MS data were processed using the Analyst (v1.6.3, AB SCIEX, USA) and then indentification of metabolites was conducted by match of mass spectrum to reference library of the local MetWare database (MWDB) based on the standard compounds or public databases including METLIN [118]. The secondary spectrum and retention time (RT) of the metabolites in the project samples were compared with MWDB, specifically, the MS tolerance and MS2 tolerance were set as 20 ppm, RT offset did not exceed 0.2 min. Quantification of metabolites were conducted using the multiple reaction monitoring (MRS), and the sub-data representing chromatographic peak areas of metabolites was subjected to log transform (log2) and mean centering for further orthogonal partial least squares-discriminant analysis (OPLS-DA) using the “OPLSR.Anal” function of the R package MetaboAnalystR [119]. In order to avoid overfitting, a permutation test (200 permutations) was performed for OPLS-DA test. Significantly regulated metabolites between groups were determined by VIP (Variable important in projection) value ≥ 1 and Log2 FC value ≥ 1 or ≤ -1.

### Electronic supplementary material

Below is the link to the electronic supplementary material.

Supplementary Material 1

Supplementary Material 2

Supplementary Material 3

Supplementary Material 4

Supplementary Material 5

## Data Availability

The authors declare that the data supporting the findings of this study are available within the article and its supplementary information files, and also available on reasonable request from the corresponding author Dianhai Hou. Furthermore, the assembled genome described in this study was deposited in GenBank under the accession JAJHTX000000000, and was associated with BioProject PRJNA642370 and BioSample SAMN22964708.

## References

[1] Riley R, Salamov AA, Brown DW, Nagy LG, Floudas D, Held BW (2014). Extensive sampling of basidiomycete genomes demonstrates inadequacy of the white-rot/brown-rot paradigm for wood decay fungi. Proc Natl Acad Sci U S A.

[2] Szychowski KA, Skora B, Pomianek T, Gminski J (2021). Inonotus obliquus - from folk medicine to clinical use. J Tradit Complement Med.

[3] Balandaykin ME, Zmitrovich IV (2015). Review on Chaga medicinal mushroom, Inonotus obliquus (higher Basidiomycetes): realm of medicinal applications and approaches on estimating its resource potential. Int J Med Mushrooms.

[4] Zhong XH, Ren K, Lu SJ, Yang SY, Sun DZ (2009). Progress of research on Inonotus obliquus. Chin J Integr Med.

[5] Zhao YX, Zheng WF (2021). Deciphering the antitumoral potential of the bioactive metabolites from medicinal mushroom Inonotus obliquus. J Ethnopharmacol.

[6] Wasser S (2002). Medicinal mushrooms as a source of antitumor and immunomodulating polysaccharides. Appl Microbiol Biot.

[7] Lemieszek MK, Langner E, Kaczor J, Kandefer-Szerszen M, Sanecka B, Mazurkiewicz W (2011). Anticancer effects of fraction isolated from fruiting bodies of Chaga medicinal mushroom, Inonotus obliquus (Pers.:Fr.) Pilat (Aphyllophoromycetideae): in vitro studies. Int J Med Mushrooms.

[8] Song Y, Hui J, Kou W, Xin R, Jia F, Wang N (2008). Identification of Inonotus obliquus and analysis of antioxidation and antitumor activities of polysaccharides. Curr Microbio.

[9] Glamoclija J, Ciric A, Nikolic M, Fernandes A, Barros L, Calhelha RC (2015). Chemical characterization and biological activity of Chaga (Inonotus obliquus), a medicinal mushroom. J Ethnopharmacol.

[10] Lee MG, Kwon YS, Nam KS, Kim SY, Hwang IH, Kim S (2021). Chaga mushroom extract induces autophagy via the AMPK-mTOR signaling pathway in breast cancer cells. J Ethnopharmacol.

[11] Handa N, Yamada T, Tanaka R (2012). Four new lanostane-type triterpenoids from Inonotus obliquus. Phytochem Lett.

[12] Zhao FQ, Xia GY, Chen LX, Zhao JL, Xie ZF, Qiu F (2016). Chemical constituents from Inonotus obliquus and their antitumor activities. J Nat Med.

[13] Song FQ, Liu Y, Kong XS, Chang W, Song G (2013). Progress on understanding the anticancer mechanisms of medicinal mushroom: Inonotus obliquus. Asian Pac J Cancer Prev.

[14] Saar M (1991). Fungi in khanty folk medicine. J Ethnopharmacol.

[15] Zheng WF, Gu Q, Chen CF, Yang SZ, Chu CC (2007). Aminophenols and mold-water-extracts affect the accumulation of flavonoids and their antioxidant activity in cultured mycelia of Inonotus obliquus. Mycosystema.

[16] Staniszewska J, Szymański M, Ignatowicz E (2017). Antitumor and immunomodulatory activity of Inonotus obliquus. Herba Pol.

[17] Burmasova MA, Utebaeva AA, Sysoeva EV, Sysoeva MA (2019). Melanins of Inonotus obliquus: bifidogenic and antioxidant properties. Biomolecules.

[18] Wang Q, Mu H, Zhang L, Dong D, Zhang W, Duan J (2015). Characterization of two water-soluble lignin metabolites with antiproliferative activities from Inonotus obliquus. Int J Biol Macromol.

[19] Nakajima Y, Sato Y, Konishi T (2007). Antioxidant small phenolic ingredients in Inonotus obliquus (persoon) Pilat (Chaga). Chem Pharm Bull.

[20] Zheng W, Zhang M, Zhao Y, Wang Y, Miao K, Wei Z (2009). Accumulation of antioxidant phenolic constituents in submerged cultures of Inonotus obliquus. Bioresour Technol.

[21] Zheng W, Zhang M, Zhao Y, Miao K, Pan S, Cao F (2011). Analysis of antioxidant metabolites by solvent extraction from sclerotia of Inonotus obliquus (Chaga). Phytochem Anal.

[22] Zheng W, Miao K, Liu Y, Zhao Y, Zhang M, Pan S (2010). Chemical diversity of biologically active metabolites in the sclerotia of Inonotus obliquus and submerged culture strategies for up-regulating their production. Appl Microbiol Biotechnol.

[23] Zheng W, Zhang M, Zhao Y, Miao K, Jiang H (2009). NMR-based metabonomic analysis on effect of light on production of antioxidant phenolic compounds in submerged cultures of Inonotus obliquus. Bioresour Technol.

[24] Zheng W, Miao K, Zhang Y, Pan S, Zhang M, Jiang H (2009). Nitric oxide mediates the fungal-elicitor-enhanced biosynthesis of antioxidant polyphenols in submerged cultures of Inonotus obliquus. Microbiology.

[25] Zheng W, Zhao Y, Zheng X, Liu Y, Pan S, Dai Y (2011). Production of antioxidant and antitumor metabolites by submerged cultures of Inonotus obliquus cocultured with Phellinus punctatus. Appl Microbiol Biotechnol.

[26] Zheng W, Zhao Y, Zhang M, Wei Z, Miao K, Sun W (2009). Oxidative stress response of Inonotus obliquus induced by hydrogen peroxide. Med Mycol.

[27] Duan Y, Han H, Qi J, Gao JM, Xu Z, Wang P (2022). Genome sequencing of Inonotus obliquus reveals insights into candidate genes involved in secondary metabolite biosynthesis. BMC Genomics.

[28] Fradj N, Goncalves Dos Santos KC, de Montigny N, Awwad F, Boumghar Y, Germain H (2019). RNA-Seq de novo assembly and differential transcriptome analysis of Chaga (Inonotus obliquus) cultured with different betulin sources and the regulation of genes involved in terpenoid biosynthesis. Int J Mol Sci.

[29] Gotthardt M, Kanawati B, Schmidt F, Asam S, Hammerl R, Frank O (2020). Comprehensive analysis of the alternaria mycobolome using mass spectrometry based metabolomics. Mol Nutr Food Res.

[30] Griffiths WJ, Koal T, Wang Y, Kohl M, Enot DP, Deigner HP (2010). Targeted metabolomics for biomarker discovery. Angew Chem Int Ed Engl.

[31] Chen W, Gong L, Guo Z, Wang W, Zhang H, Liu X (2013). A novel integrated method for large-scale detection, identification, and quantification of widely targeted metabolites: application in the study of rice metabolomics. Mol Plant.

[32] Ohta E, Nakayama Y, Mukai Y, Bamba T, Fukusaki E (2016). Metabolomic approach for improving ethanol stress tolerance in Saccharomyces cerevisiae. J Biosci Bioeng.

[33] Yang Y, Yang J, Wang H, Jin Y, Liu J, Jia R (2021). Analysis of primary metabolites of Morchella fruit bodies and mycelium based on widely targeted metabolomics. Arch Microbiol.

[34] Yang M, Zhao Y, Qin Y, Xu R, Yang Z, Peng H (2021). Untargeted metabolomics and targeted quantitative qnalysis of temporal and spatial variations in specialized metabolites accumulation in Poria cocos (Schw.) Wolf (Fushen). Front Plant Sci.

[35] Liu Y, Meng F, Tang P, Huang D, Li Q, Lin M (2022). Widely targeted metabolomics analysis of the changes to key non-volatile taste components in Stropharia rugosoannulata under different drying methods. Front Nutr.

[36] Yang G, Liang K, Zhou Z, Wang X, Huang G (2020). UPLC-ESI-MS/MS-based widely targeted metabolomics analysis of wood metabolites in Teak (Tectona grandis). Molecules.

[37] Yang R, Li Y, Zhang Y, Huang J, Liu J, Lin Z (2021). Widely targeted metabolomics analysis reveals key quality-related metabolites in kernels of sweet corn. Int J Genomics.

[38] Xu J, Yu X, Ye H, Gao S, Deng N, Lu Y (2021). Comparative metabolomics and proteomics reveal Vibrio parahaemolyticus targets hypoxia-related signaling pathways of Takifugu obscurus. Front Immunol.

[39] Sinclair CS, Richmond RH, Ostrander GK (2007). Characterization of the telomere regions of scleractinian coral, Acropora surculosa. Genetica.

[40] Schulmeister A, Schmid M, Thompson EM (2007). Phosphorylation of the histone H3.3 variant in mitosis and meiosis of the urochordate Oikopleura dioica. Chromosome Res.

[41] Teixeira MT, Gilson E (2005). Telomere maintenance, function and evolution: the yeast paradigm. Chromosome Res.

[42] Weiss H, Scherthan H (2002). Aloe spp.--plants with vertebrate-like telomeric sequences. Chromosome Res.

[43] Nierman WC, Pain A, Anderson MJ, Wortman JR, Kim HS, Arroyo J (2005). Genomic sequence of the pathogenic and allergenic filamentous fungus aspergillus fumigatus. Nature.

[44] Forney JD, Blackburn EH (1988). Developmentally controlled telomere addition in wild-type and mutant paramecia. Mol Cell Biol.

[45] Robey MT, Caesar LK, Drott MT, Keller NP, Kelleher NL (2021). An interpreted atlas of biosynthetic gene clusters from 1,000 fungal genomes. Proc Natl Acad Sci U S A.

[46] Zhong JJ, Xiao JH. Secondary metabolites from higher Fungi: Discovery, Bioactivity, and Bioproduction. In: Zhong JJ, Bai FW, Zhang W, editors. Biotechnology in China I: from Bioreaction to Bioseparation and Bioremediation. Springer: Gemany,; 2009. pp. 79–150. 10.1007/10_2008_26 19475376

[47] Hautbergue T, Jamin EL, Debrauwer L, Puel O, Oswald IP (2018). From genomics to metabolomics, moving toward an integrated strategy for the discovery of fungal secondary metabolites. Nat Prod Rep.

[48] Schüffler A, Anke T, Schüffler A (2018). Secondary metabolites of Basidiomycetes. Physiology and Genetics: selected Basic and Applied aspects.

[49] Macheleidt J, Mattern DJ, Fischer J, Netzker T, Weber J, Schroeckh V (2016). Regulation and role of fungal secondary metabolites. Annu Rev Genet.

[50] Yan L, Zhao H, Zhao X, Xu X, Di Y, Jiang C (2018). Production of bioproducts by endophytic fungi: chemical ecology, biotechnological applications, bottlenecks, and solutions. Appl Microbiol Biotechnol.

[51] Lingham RB, Silverman KC, Jayasuriya H, Kim BM, Amo SE, Wilson FR (1998). Clavaric acid and steroidal analogues as ras- and FPP-directed inhibitors of human farnesyl-protein transferase. J Med Chem.

[52] Godio RP, Martin JF (2009). Modified oxidosqualene cyclases in the formation of bioactive secondary metabolites: biosynthesis of the antitumor clavaric acid. Fungal Genet Biol.

[53] Zhang Y, Gao W, Sonnenberg A, Chen Q, Zhang J, Huang C (2021). Genetic linkage and physical mapping for an oyster mushroom Pleurotus cornucopiae and QTL analysis for the trait cap color. Appl Environ Microbiol.

[54] Zhu YJ, Xu J, Sun C, Zhou SG, Xu HB, Nelson DR (2015). Chromosome-level genome map provides insights into diverse defense mechanisms in the medicinal fungus Ganoderma sinense. Sci Rep.

[55] Chung CL, Lee TJ, Akiba M, Lee HH, Kuo TH, Liu D (2017). Comparative and population genomic landscape of Phellinus noxius: a hypervariable fungus causing root rot in trees. Mol Ecol.

[56] Lang D, Zhang S, Ren P, Liang F, Sun Z, Meng G (2020). Comparison of the two up-to-date sequencing technologies for genome assembly: HiFi reads of Pacific Biosciences Sequel II system and ultralong reads of Oxford Nanopore. Gigascience.

[57] Lafontaine DL, Yang L, Dekker J, Gibcus JH (2021). Hi-C 3.0: improved protocol for genome-wide chromosome conformation capture. Curr Protoc.

[58] Lieberman-Aiden E, van Berkum NL, Williams L, Imakaev M, Ragoczy T, Telling A (2009). Comprehensive mapping of long-range interactions reveals folding principles of the human genome. Science.

[59] Lin Y, Jain R, Yan Y (2014). Microbial production of antioxidant food ingredients via metabolic engineering. Curr Opin Biotechnol.

[60] Olsen KM, Lea US, Slimestad R, Verheul M, Lillo C (2008). Differential expression of four Arabidopsis PAL genes; PAL1 and PAL2 have functional specialization in abiotic environmental-triggered flavonoid synthesis. J Plant Physiol.

[61] Gil-Ramírez A, Pavo-Caballero C, Baeza E, Baenas N, Garcia-Viguera C, Marín FR (2016). Mushrooms do not contain flavonoids. J Funct Foods.

[62] Liu C, Kakeya H (2020). Cryptic chemical communication: secondary metabolic responses revealed by microbial co-culture. Chem Asian J.

[63] Zhu L, Xu X (2013). Stimulatory effect of different lignocellulosic materials for phenolic compound production and antioxidant activity from Inonotus obliquus in submerged fermentation. Appl Biochem Biotechnol.

[64] Xu X, Hu Y, Quan L (2014). Production of bioactive polysaccharides by Inonotus obliquus under submerged fermentation supplemented with lignocellulosic biomass and their antioxidant activity. Bioprocess Biosyst Eng.

[65] Xiang Y, Xu X, Li J (2012). Chemical properties and antioxidant activity of exopolysaccharides fractions from mycelial culture of Inonotus obliquus in a ground corn stover medium. Food Chem.

[66] Mishra SK, Kang JH, Kim DK, Oh SH, Kim MK (2012). Orally administered aqueous extract of Inonotus obliquus ameliorates acute inflammation in dextran sulfate sodium (DSS)-induced colitis in mice. J Ethnopharmacol.

[67] Du X, Mu H, Zhou S, Zhang Y, Zhu X (2013). Chemical analysis and antioxidant activity of polysaccharides extracted from Inonotus obliquus sclerotia. Int J Biol Macromol.

[68] Zou G, Li B, Wang Y, Yin X, Gong M, Shang JJ (2021). Efficient conversion of spent mushroom substrate into a high value-added anticancer drug pentostatin with engineered Cordyceps militaris. Green Chem.

[69] Zhang H, Li Z, Zhou S, Li SM, Ran H, Song Z (2022). A fungal NRPS-PKS enzyme catalyses the formation of the flavonoid naringenin. Nat Commun.

[70] Xia YL, Luo FF, Shang YF, Chen PL, Lu YZ, Wang CS (2017). Fungal cordycepin biosynthesis is coupled with the production of the safeguard molecule pentostatin. Cell Chem Biol.

[71] Saghai-Maroof MA, Soliman KM, Jorgensen RA, Allard RW (1984). Ribosomal DNA spacer-length polymorphisms in barley: mendelian inheritance, chromosomal location, and population dynamics. Proc Natl Acad Sci U S A.

[72] Lin WP, Shi YH, Jia GT, Sun HY, Sun TY, Hou DH (2021). Genome sequencing and annotation and phylogenomic analysis of the medicinal mushroom Amauroderma rugosum, a traditional medicinal species in the family Ganodermataceae. Mycologia.

[73] Lin WP, Jia GT, Sun HY, Sun TY, Hou DH (2020). Genome sequence of the fungus pycnoporus sanguineus, which produces cinnabarinic acid and pH- and thermo-stable laccases. Gene.

[74] Wenger AM, Peluso P, Rowell WJ, Chang PC, Hall RJ, Concepcion GT (2019). Accurate circular consensus long-read sequencing improves variant detection and assembly of a human genome. Nat Biotechnol.

[75] Cheng HY, Concepcion GT, Feng XW, Zhang HW, Li H (2021). Haplotype-resolved de novo assembly using phased assembly graphs with hifiasm. Nat Methods.

[76] Walker BJ, Abeel T, Shea T, Priest M, Abouelliel A, Sakthikumar S et al. Pilon: an integrated tool for comprehensive microbial variant detection and genome assembly improvement. PLoS ONE. 2014;9: e112963. 10.1371/journal.pone.0112963 PMC4237348 25409509

[77] McGinnis S, Madden TL (2004). BLAST: at the core of a powerful and diverse set of sequence analysis tools. Nucleic Acids Res.

[78] Birney E, Clamp M, Durbin R (2004). GeneWise and Genomewise. Genome Res.

[79] Haas BJ, Papanicolaou A, Yassour M, Grabherr M, Blood PD, Bowden J (2013). De novo transcript sequence reconstruction from RNA-seq using the Trinity platform for reference generation and analysis. Nat Protoc.

[80] Grabherr MG, Haas BJ, Yassour M, Levin JZ, Thompson DA, Amit I (2011). Full-length transcriptome assembly from RNA-Seq data without a reference genome. Nat Biotechnol.

[81] Trapnell C, Williams BA, Pertea G, Mortazavi A, Kwan G, van Baren MJ (2010). Transcript assembly and quantification by RNA-Seq reveals unannotated transcripts and isoform switching during cell differentiation. Nat Biotechnol.

[82] Stanke M, Steinkamp R, Waack S, Morgenstern B (2004). AUGUSTUS: a web server for gene finding in eukaryotes. Nucleic Acids Res.

[83] Ter-Hovhannisyan V, Lomsadze A, Chernoff YO, Borodovsky M (2008). Gene prediction in novel fungal genomes using an ab initio algorithm with unsupervised training. Genome Res.

[84] Haas BJ, Salzberg SL, Zhu W, Pertea M, Allen JE, Orvis J (2008). Automated eukaryotic gene structure annotation using EVidenceModeler and the program to assemble spliced alignments. Genome Biol.

[85] Chan PP, Lowe TM (2019). tRNAscan-SE: searching for tRNA genes in genomic sequences. Methods Mol Biol.

[86] Lagesen K, Hallin P, Rodland EA, Staerfeldt HH, Rognes T, Ussery DW (2007). RNAmmer: consistent and rapid annotation of ribosomal RNA genes. Nucleic Acids Res.

[87] Nawrocki EP, Eddy SR (2013). Infernal 1.1: 100-fold faster RNA homology searches. Bioinformatics.

[88] Griffiths-Jones S, Moxon S, Marshall M, Khanna A, Eddy SR, Bateman A (2005). Rfam: annotating non-coding RNAs in complete genomes. Nucleic Acids Res.

[89] Tarailo-Graovac M, Chen N. Using RepeatMasker to identify repetitive elements in genomic sequences. Curr Protoc Bioinformatics. 2009, 25:4.10.1–14. 10.1002/0471250953.bi0410s25 19274634

[90] Bao W, Kojima KK, Kohany O (2015). Repbase Update, a database of repetitive elements in eukaryotic genomes. Mob DNA.

[91] Benson G (1999). Tandem repeats finder: a program to analyze DNA sequences. Nucleic Acids Res.

[92] Katoh K, Rozewicki J, Yamada KD (2019). MAFFT online service: multiple sequence alignment, interactive sequence choice and visualization. Brief Bioinform.

[93] Nakamura T, Yamada KD, Tomii K, Katoh K (2018). Parallelization of MAFFT for large-scale multiple sequence alignments. Bioinformatics.

[94] Crooks GE, Hon G, Chandonia JM, Brenner SE (2004). WebLogo: a sequence logo generator. Genome Res.

[95] Ashburner M, Ball CA, Blake JA, Botstein D, Butler H, Cherry JM (2000). Gene ontology: tool for the unification of biology. Nat Genet.

[96] Kanehisa M, Goto S, Sato Y, Furumichi M, Tanabe M (2012). KEGG for integration and interpretation of large-scale molecular data sets. Nucleic Acids Res.

[97] Galperin MY, Wolf YI, Makarova KS, Vera Alvarez R, Landsman D, Koonin EV (2021). COG database update: focus on microbial diversity, model organisms, and widespread pathogens. Nucleic Acids Res.

[98] Boeckmann B, Bairoch A, Apweiler R, Blatter MC, Estreicher A, Gasteiger E (2003). The SWISS-PROT protein knowledgebase and its supplement TrEMBL in 2003. Nucleic Acids Res.

[99] El-Gebali S, Mistry J, Bateman A, Eddy SR, Luciani A, Potter SC (2019). The pfam protein families database in 2019. Nucleic Acids Res.

[100] Eddy SR (2009). A new generation of homology search tools based on probabilistic inference. Genome Inf.

[101] Simao FA, Waterhouse RM, Ioannidis P, Kriventseva EV, Zdobnov EM (2015). BUSCO: assessing genome assembly and annotation completeness with single-copy orthologs. Bioinformatics.

[102] Zhang H, Yohe T, Huang L, Entwistle S, Wu P, Yang Z (2018). dbCAN2: a meta server for automated carbohydrate-active enzyme annotation. Nucleic Acids Res.

[103] Buchfink B, Xie C, Huson DH (2015). Fast and sensitive protein alignment using DIAMOND. Nat Methods.

[104] Xu J, Zhang H, Zheng J, Dovoedo P, Yin Y (2020). eCAMI: simultaneous classification and motif identification for enzyme annotation. Bioinformatics.

[105] Lombard V, Golaconda Ramulu H, Drula E, Coutinho PM, Henrissat B (2013). The carbohydrate-active enzymes database (CAZy) in 2013. Nucleic Acids Res.

[106] Blin K, Shaw S, Kloosterman AM, Charlop-Powers Z, van Wezel GP, Medema MH (2021). antiSMASH 6.0: improving cluster detection and comparison capabilities. Nucleic Acids Res.

[107] Bolger AM, Lohse M, Usadel B (2014). Trimmomatic: a flexible trimmer for Illumina sequence data. Bioinformatics.

[108] Kim D, Langmead B, Salzberg SL (2015). HISAT: a fast spliced aligner with low memory requirements. Nat Methods.

[109] Anders S, Pyl PT, Huber W (2015). HTSeq–a Python framework to work with high-throughput sequencing data. Bioinformatics.

[110] Roberts A, Trapnell C, Donaghey J, Rinn JL, Pachter L (2011). Improving RNA-Seq expression estimates by correcting for fragment bias. Genome Biol.

[111] Anders S, Huber W (2012). Differential expression of RNA-Seq data at the gene level–the DESeq package.

[112] Sheynkman GM, Shortreed MR, Frey BL, Smith LM (2013). Discovery and mass spectrometric analysis of novel splice-junction peptides using RNA-Seq. Mol Cell Proteomics.

[113] Wisniewski JR, Zougman A, Nagaraj N, Mann M (2009). Universal sample preparation method for proteome analysis. Nat Methods.

[114] Dayon L, Hainard A, Licker V, Turck N, Kuhn K, Hochstrasser DF (2008). Relative quantification of proteins in human cerebrospinal fluids by MS/MS using 6-plex isobaric tags. Anal Chem.

[115] Xing L, Yuan C, Wang M, Lin Z, Shen B, Hu Z (2017). Dynamics of the Interaction between Cotton Bollworm Helicoverpa armigera and Nucleopolyhedrovirus as revealed by Integrated Transcriptomic and proteomic analyses. Mol Cell Proteomics.

[116] Marcin K (2009). What is strong correlation?. Teach Stat.

[117] Akoglu H (2018). User’s guide to correlation coefficients. Turk J Emerg Med.

[118] Tautenhahn R, Cho K, Uritboonthai W, Zhu ZJ, Patti GJ, Siuzdak G (2012). An accelerated workflow for untargeted metabolomics using the METLIN database. Nat Biotechnol.

[119] Chong J, Xia J (2018). MetaboAnalystR: an R package for flexible and reproducible analysis of metabolomics data. Bioinformatics.

