# Supplementary material for: Integrated omic profiling of the medicinal mushroom Inonotus obliquus under submerged conditions
Source: BMC Genomics. 2023 Sep 19;24:554. doi: 10.1186/s12864-023-09656-z (PMC10507853; doi:10.1186/s12864-023-09656-z)
Supplement: Supplementary file 1 — Supplementary Material 1 [file 12864_2023_9656_MOESM1_ESM.docx]

**Table S1** Statistics of the Illumina HiSeq X Ten sequencing data of the *Inonotus obliquus* CFCC 83414.

| **No. of raw reads** | **Raw bases (Gbp)** | **Raw Q30** ^1^ | **Raw GC** ^2^ | **No. of clean reads** ^3^ | **Clean bases (Gbp)** ^3^ | **Clean Q30** ^1^ | **Clean GC** ^2^ | **Valid bases** ^4^ | **Duplicate rate** |
| --- | --- | --- | --- | --- | --- | --- | --- | --- | --- |
| 63.28M | 9.49 | 94.88% | 44.50 | 59.19M | 8.82G | 96.76% | 44.34 | 92.91% | 7.09% |

1. Raw/clean Q30: percentage of bases with Qphred (Phred score) >30 (error rate < 0.001) in raw/clean bases;
2. Raw/clean GC: percentage of GC bases in raw/clean bases;
3. Clean reads/bases: number of reads/bases in the resulted sequence data after filtering adaptors, low quality reads, and trim ambiguous bases from 5' and/or end of reads from raw data;

4. Valid bases: percentage of clean reads in raw reads.

**Table S2** Statistics of the PacBio Sequel II sequencing data of the *Inonotus obliquus* CFCC 83414.

| **Sample** | **No. of reads** | **Total length (Gbp)** | **Length of reads (bp)** | | | | | |
| --- | --- | --- | --- | --- | --- | --- | --- | --- |
|  |  |  | **N50** | **N90** | **Mean** | **Median** | **Maximum** | **Minimum** |
| HIFI reads | 232,212 | 2.32 | 11,001 | 7,551 | 9,985 | 10,038 | 41,322 | 505 |

**Table S3** BUSCO analysis on the assembly and annotation of the *Inonotus obliquus* CFCC 83414*.*

| **Iterms** | **Number** | **Percent (%)** |
| --- | --- | --- |
| Complete BUSCOs | 276 | 95.2 |
| Complete and single-copy BUSCOs | 275 | 94.83 |
| Complete and duplicated BUSCOs | 1 | 0.34 |
| Fragmented BUSCOs | 9 | 3.1 |
| Missing BUSCOs | 5 | 1.7 |
| Total BUSCO groups searched | 290 | 100 |

**Table S4** Statistics of contigs of the *Inonotus obliquus* CFCC 83414.

| **Contig No.** | **Size** | **GC content (%)** | **Contig No.** | **Size** | **GC content (%)** |
| --- | --- | --- | --- | --- | --- |
| 01 | 4,357,162 | 48.08 | 17 | 34,712 | 26.16 |
| 02 | 4,356,680 | 47.96 | 18 | 33,983 | 26.23 |
| 03 | 3,807,492 | 47.56 | 19 | 30,789 | 26.30 |
| 04 | 3,182,639 | 48.00 | 20 | 26,618 | 24.36 |
| 05 | 3,094,001 | 48.04 | 21 | 25,414 | 25.44 |
| 06 | 2,980,324 | 47.72 | 22 | 21,608 | 24.00 |
| 07 | 2,398,419 | 47.62 | 23 | 20,945 | 26.17 |
| 08 | 2,056,250 | 47.44 | 24 | 20,186 | 47.42 |
| 09 | 2,017,917 | 47.53 | 25 | 19,510 | 24.61 |
| 10 | 1,942,692 | 47.37 | 26 | 19,295 | 24.96 |
| 11 | 1,935,300 | 47.51 | 27 | 19,102 | 23.47 |
| 12 | 1,920,097 | 47.59 | 28 | 18,630 | 24.91 |
| 13 | 1,582,910 | 47.52 | 29 | 18,362 | 24.11 |
| 14 | 55,104 | 24.07 | 30 | 18,313 | 23.67 |
| 15 | 43,145 | 25.27 | 31 | 16,440 | 48.53 |
| 16 | 38,208 | 47.38 | 32 | 15,401 | 22.80 |

**Table S5** Statistics of annotations of 8352 genes of the *Inonotus obliquus* CFCC 83414.

| **Annotation Database** | **Annotated number** | **Annotated ratio** |
| --- | --- | --- |
| GO | 3,674 | 43% |
| KEGG | 2,977 | 35% |
| COG | 3,885 | 46% |
| NR | 7,897 | 94% |
| TrEMBL | 7,551 | 90% |
| Swissprot | 4,651 | 55% |
| Pfam | 5,654 | 67% |
| Total | 7,915 | 94% |

**Table S6** Overview of the carbohydrate-active enzymes (CAZymes) identified in the *Inonotus obliquus* CFCC 83414.

| **Category** | **Number** | **Superfamily number** |
| --- | --- | --- |
| Carbohydrate-Binding Modules (CBMs) | 3 | 2 |
| [Glycoside Hydrolases (GHs)](http://www.cazy.org/Glycoside-Hydrolases.html) | 187 | 47 |
| [Glycosyl Transferases (GTs)](http://www.cazy.org/GlycosylTransferases.html) | 69 | 29 |
| [Polysaccharide Lyases (PLs)](http://www.cazy.org/Polysaccharide-Lyases.html) | 13 | 6 |
| [Carbohydrate Esterases (CEs)](http://www.cazy.org/Carbohydrate-Esterases.html) | 21 | 8 |
| [Auxiliary Activities (AAs)](http://www.cazy.org/Auxiliary-Activities.html) | 72 | 9 |

**Table S7** Identification of 19 secondary metabolite BGCs of the *Inonotus obliquus* CFCC 83414.

| **No.** | **Location** | | | **Type ^1^** | **Similar BGC** |
| --- | --- | --- | --- | --- | --- |
|  | **Contig** | **Start** | **End** |  |  |
| 1 | 1 | 3,731,526 | 3,752,530 | Terpene | None |
| 2 | 1 | 3,777,685 | 3,795,388 | Terpene | None |
| 3 | 1 | 3,802,601 | 3,826,190 | Terpene | None |
| 4 | 1 | 3,828,053 | 3,849,189 | Terpene | None |
| 5 | 2 | 934,598 | 978,498 | T1PKS, NRPS-like | None |
| 6 | 2 | 1,110,412 | 1,158,935 | T1PKS, NRPS-like | None |
| 7 | 2 | 1,353,095 | 1,376,630 | Terpene | None |
| 8 | 2 | 2,897,461 | 2,945,434 | NRPS | None |
| 9 | 3 | 124,216 | 177,147 | Terpene | None |
| 10 | 3 | 379,986 | 414,806 | NRPS-like | None |
| 11 | 3 | 577,402 | 622,365 | NRPS-like | None |
| 12 | 3 | 670,184 | 683,787 | Terpene | None |
| 13 | 3 | 2,572,719 | 2,596,622 | Terpene | Clavaric acid |
| 14 | 5 | 122,427 | 172,672 | T1PKS | None |
| 15 | 5 | 1,523,651 | 1,544,845 | Terpene | None |
| 16 | 7 | 820,157 | 832,276 | Terpene | None |
| 17 | 7 | 1,523,651 | 1,544,845 | Terpene | None |
| 18 | 11 | 1,578,205 | 1,599,682 | Terpene | None |
| 19 | 12 | 154,405 | 198,786 | NRPS-like | None |

^1^ PKS: polyketide synthase; NRPS: non-ribosomal peptide synthetase.

**Table S8** Overview of the quality of filtered sequence data in differential transcriptomic analysis of the *Inonotus obliquus* CFCC 83414.

| **Sample** | **Raw**  **reads** ^1^ | **Raw**  **bases** ^1^ | **Clean**  **reads** ^2^ | **Clean**  **bases** ^2^ | **Valid bases** | **Q30** ^3^ | **GC (%)** |
| --- | --- | --- | --- | --- | --- | --- | --- |
| SEC1 | 53.69M | 8.05G | 52.70M | 7.38G | 91.64% | 94.30% | 50.67% |
| SEC2 | 51.22M | 7.68G | 50.32M | 7.08G | 92.14% | 94.50% | 50.64% |
| SEC3 | 49.96M | 7.49G | 49.07M | 6.92G | 92.33% | 94.51% | 50.78% |
| FEC1 | 46.67M | 7.00G | 45.89M | 6.50G | 92.78% | 94.59% | 50.85% |
| FEC2 | 50.44M | 7.57G | 49.59M | 7.01G | 92.64% | 94.60% | 50.92% |
| FEC3 | 52.47M | 7.87G | 51.59M | 7.29G | 92.60% | 94.60% | 50.90% |

1. Raw reads/bases: number of reads/bases in original sequence data (raw data);
2. Clean reads/bases: number of reads/bases in the resulted sequence data after filtering adaptors, low quality reads, and trim ambiguous bases from 5' and/or end of reads from raw data;

3. Q30 (%): percentage of bases with Qphred (Phred score) >30 (error rate < 0.001) in raw bases.

**Table S9 Statistics of transcriptomic reads mapped to the reference genome of the *Inonotus obliquus* CFCC 83414.**

| **Iterm** | **SEC1** | **SEC2** | **SEC3** | **FEC1** | **FEC2** | **FEC3** |
| --- | --- | --- | --- | --- | --- | --- |
| No. of total clean reads | 52,703,352 | 50,322,130 | 49,071,746 | 45,892,596 | 49,594,590 | 51,591,200 |
| Total mapped ^1^ | 51160992 (97.07%) | 48,788,489 (96.95%) | 47,565,243 (96.93%) | 44,528,798 (97.03%) | 48,082,154 (96.95%) | 50,015,317 (96.95%) |
| Multiple mapped ^2^ | 3,911,379 (7.42%) | 3,750,413 (7.45%) | 1,343,890 (2.74%) | 2,392,622 (5.21%) | 1,108,917 (2.24%) | 1,039,841 (2.02%) |
| Uniquely mapped ^3^ | 47,249,613 (89.65%) | 45,038,076 (89.50%) | 46,221,353 (94.19%) | 42,136,176 (91.81%) | 46,973,237 (94.71%) | 48,975,476 (94.93%) |
| Reads map to '+' ^4^ | 23,616,668 (44.81%) | 22,510,770 (44.73%) | 23,101,073 (47.08%) | 21,059,687 (45.89%) | 23,480,236 (47.34%) | 24,478,611 (47.45%) |
| Reads map to '-' ^4^ | 23,632,945 (44.84%) | 22,527,306 (44.77%) | 23,120,280 (47.12%) | 21,076,489 (45.93%) | 23,493,001 (47.37%) | 24,496,865 (47.48%) |
| Non-splice reads | 28,617,545 (54.30%) | 27,080,828 (53.81%) | 27,800,994 (56.65%) | 25,107,135 (54.71%) | 27,996,266 (56.45%) | 29,211,524 (56.62%) |
| Splice reads ^5^ | 18,632,068 (35.35%) | 17,957,248 (35.68%) | 18,420,359 (37.54%) | 17,029,041 (37.11%) | 18,976,971 (38.26%) | 19,763,952 (38.31%) |
| Detected genes ^6^ | 8095 | 8102 | 8092 | 8043 | 8074 | 8049 |

1. Total mapped: No. of reads mapped to the reference genome;

2. Multiple mapped: No. of reads mapped to multiple sites on the reference genome;

3. Uniquely mapped: No. of reads mapped to the unique sites on the reference genome;

4: Reads map to '+'/'-': No. of reads uniquely mapped to the positive/negative strand of the reference genome;
5. Splice reads: uniquely mapped reads linking two exons;

6. Detected genes: genes with reads mapped.
